# Pre-transport risk factors for severity of respiratory associated symptoms in unweaned calves following long-distance transport

**DOI:** 10.3389/fvets.2026.1760080

**Published:** 2026-02-23

**Authors:** Luca L. van Dijk, Susanne Siegmann, Niamh L. Field, Katie Sugrue, Cornelis G. van Reenen, Eddie A. M. Bokkers, Gearoid Sayers, Muireann Conneely

**Affiliations:** 1 Teagasc, Animal and Grassland Research and Innovation Centre, Fermoy, Ireland; 2 Department of Biological and Pharmaceutical Sciences, Munster Technological University, Tralee, Ireland; 3 Animal Production Systems Group, Wageningen University and Research, Wageningen, Netherlands

**Keywords:** bovine respiratory disease, calf transport, respiratory disease, risk factor, thoracic ultrasound

## Abstract

An essential component of protecting calf welfare during transport is identifying those most likely to develop transport-related respiratory disease. We aimed to associate pre-transport risk factors with post-transport respiratory disease based on the highest recorded value for thoracic ultrasound score, clinical respiratory score, neutrophil, monocyte, white blood cell count, neutrophil: lymphocyte ratio, and lowest recorded value for lymphocyte count and total immunoglobulins between arrival and 3-weeks post-arrival, in calves deemed legally fit for transport. The highest or lowest point of an individual variable within 3 weeks post-arrival was chosen to reflect the point at which the disease was perceived to be at its worst, and the direction of change (highest vs. lowest) was based on the reported direction of change in individual variables in calves diagnosed with respiratory disease in prior research. We studied two transport cohorts (in April and May 2022) between Ireland and The Netherlands and used generalized linear mixed models to analyze associations between precipitating health factors [pre-transport thoracic ultrasound (TUSpre), pre-transport clinical respiratory score (CRSpre)] and predisposing genetic and environmental factors (breed, sex, transport cohort, source, age, and body weight) on post-transport respiratory disease signs. Calves with a favorable TUSpre of 0 had lower post-transport ultrasound scores, neutrophil counts, and neutrophil: lymphocyte ratios than calves with a TUSpre of 2 (*p* < 0.01, *p* = 0.04, and *p* = 0.02 respectively), but CRSpre did not affect post-transport respiratory disease signs (all *p* > 0.2). Among predisposing factors, Holstein-Friesian calves developed higher ultrasound and clinical respiratory scores (showing more signs of pathology), as well as neutrophilia, monocytosis and higher neutrophil: lymphocyte ratios post-transport than Holstein-Friesian*Beef calves (*p* = 0.02, *p* = 0.01, *p* = 0.04, *p* = 0.03, and *p* = 0.01 respectively). Lighter calves experienced post-transport neutrophilia and higher neutrophil: lymphocyte ratios than heavier calves (both *p* < 0.01). We found slight increases in respiratory disease signs in cohort 1 calves, females, and calves originating from livestock marts, but these were not consistent across indicator variables. In conclusion, we should consider a high ultrasound score, dairy breed, and low body weight as risk factors for respiratory disease signs post-transport.

## 1 Introduction

Transport is a challenging stressor which many calves face early in life in modern production systems. The health and welfare consequences of transport, including suppressed immune systems and an increased risk of respiratory disease, have been documented, and those risks can persist beyond the end of transport event itself (1). Bovine respiratory disease (BRD) is a significant infectious threat for unweaned calves, causing respiratory symptoms in up to 61% of calves produced for veal, leading to an almost ubiquitous and unavoidable use of antibiotics (1). Pathogens causing BRD are commonly present on dairy farms, and commingling of calves from different farms, such as during transport, amplifies pathogen exposure. The confined trailer environment, prolonged fasting, and immune suppression can further contribute to pathogen inoculation (2, 3).

To reduce in-transport morbidity rates, fitness for transport assessments are a legal requirement in the European Union (EC 1/2005) (4), intended to ensure only healthy and well-conditioned animals are transported. In practice, this involves an observation of all animals for clinical signs of disease, amongst other physical ailments, at the point of departure, with the expectation that calves without visual clinical signs of infection will be better able to cope with the stressors of transport. However, one could argue that these assessments are not only intended to evaluate an animal’s health status at the point of departure, but also to predict whether it will remain healthy throughout the journey—particularly critical in the case of long-distance transport.

To the best of our knowledge, there have been no before-and-after studies examining the effectiveness of fitness-for-transport screening tools for unweaned non-replacement calves exposed to long-distance transport, despite these assessments being a legal requirement in Europe. It is unknown to what extent current screening tools are effective in safeguarding the health of the calf beyond the point of assessment, including those critical periods during transport and after their arrival at the destination farm.

Currently, health screening is largely focused on clinical signs of respiratory disease, including symptoms of ocular and nasal discharge, evidence of coughing, fever, among other criteria (5). Other more involved assessment options may be carried out by a veterinarian such as thoracic ultrasonography (TUS) examinations for lung lesions (which can also be performed by other trained observers), or blood-based indicators of immune function, including failure of passive transfer or elevated neutrophil and monocyte counts (6–8). These screening tools, however, are rarely used in fitness-for-transport examinations for calves. Improving a calf’s resilience to transport stressors could potentially be achieved by incorporating additional health-based risk factors such as pre-transport thoracic ultrasound scores, which have been shown to be more reliable for on-farm respiratory health screening than clinical respiratory scoring (9).

In addition to clinical health screening and thoracic ultrasonography, blood related variables may indicate infection or disease in calves. Various blood variables change during respiratory disease, neutrophilia, monocytosis, lymphopenia, and increased SAA have been observed in response to respiratory infections in calves (7, 8, 10–14), and calves with low total immunoglobulin concentrations are more at risk of developing respiratory disease (7).

It is also understood that other underlying genetic and environmental factors contribute to a calf’s health and resilience, which is of particular importance for long-distance transport. For example, adult cattle of dairy breeds appear more prone to respiratory disease compared to beef breeds (15), and male calves may have lower concentrations of protective maternal antibodies due to insufficient intake of high-quality colostrum (16), though good colostrum management can eliminate this difference in maternal antibody concentrations between male and female calves (17). The source of the calf, whether from a dairy farm or livestock mart, may also impact the severity of pathogen exposure (18), while age and body weight may correlate with a calf’s immunological maturity (19, 20).

As part of a wider research program examining calf health in relation to long distance transport from Ireland, this study aimed to examine pre-transport risk factors that influence the post-transport likelihood of signs of respiratory disease based on thoracic ultrasound, clinical assessment and blood variables, in calves deemed legally fit for transport. We hypothesized that calves without precipitating health issues would have a lower risk of post-transport respiratory disease. Additionally, we aimed to explore the effects of age, weight, breed, sex, source, and transport cohort on post-transport respiratory disease development.

## 2 Materials and methods

### 2.1 Calf selection, transport, and housing

This observational study took place in April (transport cohort 1) and May 2022 (transport cohort 2) on two commercial road-ferry-road transports of unweaned calves between commercial dairy farms and marts, via an assembly center in Ireland, and a lairage in France, to two separate veal farms in The Netherlands. We based the original sample size calculations on expected glucose values of 5.4 and 5.0 mmol/L (pre-transport and during transport, based on a 2021 pilot study) and an anticipated standard deviation of 1.0 mmol/L. Using an alpha of 0.05 and a power of 90%, we estimated a required sample size of 68 calves. Due to practical constraints, the trial included 65 calves. We repurposed the dataset for the present study, with preliminary analyses indicating clear statistical differences that justified further investigation. We also performed a post-hoc sample size analysis using values of maximum neutrophil count between arrival and 3-weeks post arrival in the current dataset, which we calculated for a TUSpre of 0 or 3. Using maximum neutrophil count means of 3.82 10^9^/L (TUSpre of 0) and 4.74 10^9^/L (TUSpre of 3), a standard deviation of 1.31 10^9^/L, alpha of 0.05, and power of 90%, we estimated a minimum required sample size of 24 calves. As our actual number of calves was larger, we deemed this dataset sufficient for further exploration.

The commercial exporter bought calves from eight commercial source dairy farms (source farm; Co. Cork, Ireland) and two commercial livestock marts (mart; Co. Cork, Ireland) where researchers enrolled them in the study. The study included all healthy Holstein-Friesian and Holstein-Friesian x beef calves over 14 days of age, regardless of sex and breed, that were presented for transport at the source farms or purchased at the mart. On average, calves were 29.1 d old (16–42 d) and weighed 56.1 kg (43–72 kg) pre-transport at the assembly center in Ireland. They consisted of Holstein-Friesian (*n* = 15) and Holstein-Friesian × Beef (beef-cross; *n* = 50) breeds and were male (*n* = 56) or female (*n* = 9). Calves spent 77 (cohort 1) to 81 (cohort 2) hours in transit, including a 16 h rest time at the assembly center in Ireland where we fed them 2 L of milk replacer (125 g/L fed at 40 °C; 21% protein, 1% fat), and a 13 h rest time at the lairage in France where we fed them 3 L of milk replacer (90 g/L fed at 40 °C; 22% protein, 19% fat). For transport cohort 1, during the ferry journey, the weather was mild with a slight overcast (15.8 °C, humidity: 72%; average data recorded by two TinyTags (Gemini Data Loggers, Chichester, United Kingdom) positioned at approximately 50 cm above floor level, on the inside wall in the front and rear of the lorry), and the sea was calm with minimal swell [mean wave height of 1.6 m at M5 buoy; (21)]. Between France and the Netherlands, the weather was mild with a slight overcast (15.9 °C, humidity: 67%). For transport cohort 2, during the ferry journey, the weather was mild with a slight overcast (17.7 °C, humidity: 66%; average data recorded by two TinyTags positioned in proximity to the lorry), and the sea was rough [mean wave height of 3.6 m at M5 buoy; (21)]. Between France and the Netherlands, there were heavy downpours (20.3 °C, humidity: 82%; average data recorded by two TinyTags positioned on the inside wall in the front and rear of the lorry). The calf selection and transit processes are described in detail by van Dijk et al. (22).

Post transport, calves were housed individually with a space allowance of 1.6 m2 per calf. The farmer fed them electrolytes in water (sodium bicarbonate–based electrolyte mix; additional ingredients not available) for the first feed and subsequently fed each calf 1.5 L of milk replacer (130 g/L mixed at 45 °C; 21% protein, 18.2% fat) twice a day in buckets. The volume of milk replacer gradually increased to 2.7 L twice a day by 3-weeks post arrival. Calves that failed to drink from a bucket were fed with floating teats. Calves were separated by stainless steel fencing and could see and touch calves in neighboring pens. More detail on the post-transport housing and management of calves is described in van Dijk et al. (23). All transport and housing conditions complied with European and Irish legislations. Calves were regularly observed by the trial team for 3 weeks, after which they were released into groups. All calves received antibiotic treatment within 24 h after arrival (Tilmicosin: 1 cc I. M.) and received an additional two courses of metaphylactic batch antibiotics in week 1 and week 2 post arrival at the veal farm (Doxycycline: 0.03 g per feed in milk twice a day for three and 5 days respectively). No calves received individual antibiotic treatments during the transport or for 3 weeks thereafter. Pre-transport individual treatments were not available.

### 2.2 Sampling

**Figure 1:**
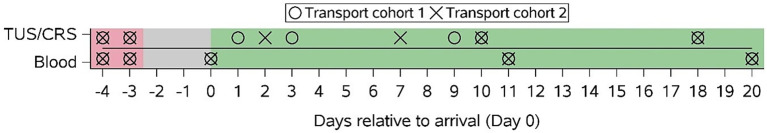
Sampling schedule of blood, thoracic ultrasound scoring (TUS), and clinical respiratory scoring (CRS) on days relative to arrival (Day 0). Sampling days are pre-transport (Days −4 and −3), during transport (Day −3 to Day 0), or on the destination veal farm (Day 0 to Day 20). Calves left the source dairy farm on Day −3. The sampling schedule shows sampling days for calves transported in transport cohorts in April and May 2022 (transport cohort 1 and 2, respectively).

The sampling schedule consisted of blood sampling, clinical respiratory scoring (CRS), and thoracic ultrasound scoring (TUS) (Figure 1). Pre-transport, calves underwent sampling (blood, CRS, and TUS) at the dairy farm of origin (*n* = 26) or at the livestock mart they were purchased at (*n* = 39). Post transport, blood sampling, and TUS and CRS scoring occurred at regular intervals; days differed slightly for transport cohorts 1 and 2 due to practical constraints (Figure 1).

Blood sampling was performed by jugular venipuncture using a 20 G, 1½ inch needle (BD Precision glide; Beckton Dickinson and Company, Franklin Lakes, NJ); we collected 26 mL of blood for every blood sample into four different BD vacutainer tubes (BD Precision glide; Beckton Dickinson and Company, Franklin Lakes, NJ) containing EDTA (6 mL), heparin (6 mL), glycolytic inhibitor (6 mL) and serum separator tubes (SST; 8.5 mL); only EDTA and SST samples contributed to this study. We based the CRS system on the Wisconsin calf health scorer (24) and adapted it for practical use in transport research (Table 1). For some calves assessed 10 Days after arrival, rectal temperature was not available due to practical constraints; rectal temperatures from 9 Days post arrival replaced these missing values. The total CRS equaled the sum of all individual clinical respiratory scores included in Table 1. We did not collect rectal temperatures pre-transport and omitted these values for the CRS calculation at this observation time. Within the Wisconsin system, a total score ≥5 or observations with two or more parameters with an individual score of 2 or 3 indicates respiratory disease (24). For our study, we expected scores to be lower due to antibiotic treatments. To improve comparative power with TUS, we considered observations with a total CRS of 0 and 1 as healthy or mild, CRS 2 or 3 as moderate and CRS ≥ 4 as severe. We blood sampled calves pre-transport (dairy farm/mart), on arrival at the veal farm, and 10- and 21-days post arrival at the veal farm. A single observer was used throughout the study and thoracic ultrasound scores and CRS were always observed at the same time.

**Table 1:** Clinical respiratory variables and point scale used for health scoring.^1^

Clinical variable	Points and description			
	0	1	2	3
Rectal temperature (°C)	<38.3	38.3–38.7	38.8–39.4	>39.4
Coughing	No cough	Single cough	Repeated cough (within ± 2 min)	–
Nasal discharge	Normal, serous discharge	Small amount of unilateral, cloudy discharge	Bilateral, cloudy, or excessive mucus	Copious, bilateral
Eye discharge	No eye discharge	Small amount of unilateral discharge (<1 cm^2^)	Moderate amount of bilateral discharge	Heavy ocular discharge
Ear position	Normal ear position	Head shaking	Slight unilateral ear droop	Bilateral ear droop

^1^Adapted from scoring systems used by McGuirk et al. (24).

We based the thoracic ultrasound scoring on a system used by Ollivett and Buczinski (6) and slightly adapted it for the current study. Prior to scanning, an observer drenched the coat over the lung area of the calf in 70% isopropyl alcohol to avoid the disturbance of ultrasound waves by trapped air. Subsequently the observer passed the ultrasound probe dorsally to ventrally within each intercostal space, progressing in a cranial direction from the 12th to the 3rd (left) or to the 1st (right) intercostal space using a 4.5–8.5 MHz ultrasound scanner (Easi-Scan veterinary ultrasound scanner, IMV Imaging; Gormanston, Ireland) set to a depth of 11 cm using a lower frequency to obtain a greater penetration (setting: late-term). The observer lifted the right front leg of the calf to scan the area of the 2nd and 1st intercostal space on the right side. We based an overall 6-tier TUS on the score of four lobes (right cranial, right caudal, left cranial, and left caudal). Score 0: Normal aerated lung in the four examined lobes. Score 1: diffuse comet-tail artefacts in one to four lobes. Score 2: Lobular or patchy consolidation in one or more lobes, yielding a theoretical total equivalence loss of functioning of less than one whole lobe. Score 3: Lobar consolidation in one lobe, or multiple lobes presenting with lobular consolidation yielding a theoretical total equivalence of loss of functioning of one whole lobe. Score 4: lobar pneumonia in two lobes. Score 5: Lobar pneumonia in three or more lobes. Thoracic ultrasound scores of 0 indicated healthy lungs, TUS of 1 indicated mild respiratory pathology, TUS of 2 indicated moderate respiratory pathology, and TUS ≥ 3 indicated severe respiratory pathology.

### 2.3 Laboratory processing

The study team refrigerated EDTA tubes directly post collection, hematology (white blood cell, neutrophil, lymphocyte, and monocyte counts) was analyzed in different locations to minimize the time between sample collection and laboratory analysis of fresh samples. For samples collected in Ireland (pre-transport), Teagasc Grange (Dunsany, Ireland) analyzed hematology samples using the Advia 2,120 system (Bayer, AG). For samples collected in the Netherlands (all other samples), Rimondia (Elspeet, the Netherlands) analyzed hematology using fluorescence flow cytometry (XT-1800i, Sysmex Europe GmbH, Germany).

We spun all SST samples at 3,000 rpm for 10 min, decanted into serum tubes, and stored in a freezer (−20 °C). Teagasc Grange (Dunsany, Ireland) analyzed all serum samples, using a commercial ELISA for immunoglobulin-A and Immunoglobulin-G (Eagle BGG69-KOI, Amherst, NH, United States), and immunoglobulin-M (Eagle BCM61-KOI, Amherst, NH, United States). Total immunoglobulins (IG) equaled the sum of immunoglobulins -A, −G, and -M.

### 2.4 Statistical analysis

Prior to statistical analysis, researchers removed data from one beef-cross calf transported as part of transport cohort 1 from the analysis due to a missing TUS observation leaving a total of 65 calves for inclusion in the analysis. Additionally, because only four calves received a TUS grade of 4, which we deemed too low for statistical analysis, we transformed these values to TUS of 3 and analyzed as part of this scoring group. We never observed thoracic ultrasound scores of 5. Thoracic ultrasound observations are commonly collapsed into two categories; the absence and presence of consolidation, and therefore collapsing any type of consolidation into the same category in this study was deemed appropriate. One monocyte value for one calf was removed as it was double the next highest value and presumed to be a fault of laboratory error.

The pre-transport risk factors examined in the analysis included precipitating respiratory health based on pre-transport thoracic ultrasound score and pre-transport clinical respiratory score, and predisposing environmental and genetic factors including breed, sex, transport cohort, source of the calf, and age and body weight at the point of departure. We performed all statistical analyses using SAS on Demand (25). For all models, we set the significance at 5% (*p* = 0.05). We used generalized linear mixed models (SAS PROC GLIMMIX) to perform statistical analyses using the following model:


$$\begin{array}{l} y=\mu +\text{TUSpre}+\text{CRSpre}+\text{Breed}+\mathrm{Sex}+\text{Transport Cohort}\\ +\text{Source}+\mathrm{Age}+\text{Weight} \end{array}$$

Dependent variables (y) were calculated as the highest value recorded after departure (from arrival to 3 weeks after) for TUS (TUSmax: score 0, 1, 2, or 3), CRS (CRSmax: score 0 to 7), neutrophil count, neutrophil: lymphocyte ratio (N/L ratio), monocyte count, and white blood cell count (WBC) or the lowest recorded value in the case of lymphocyte or IG. Expected directions of change in response to respiratory disease (highest vs. lowest value recorded) were decided based on previous research (7, 13) and were selected to reflect the point at which signs of respiratory disease were perceived to be at their worst. The model incorporated fixed effects of pre-transport TUS (TUSpre = 0, 1, 2, or 3), pre-transport CRS (CRSpre = 0, 1, 2, or 3), breed (Holstein-Friesian or beef-cross), sex (male or female), transport cohort (1 or 2), and source (source farm or mart). The model also included continuous effects of age at departure (16–42 d), and body weight at departure (43–72 kg). Preliminary analysis showed no correlation between age and weight (*R*-value: 0.18), and as a result, we did not include interaction effects between these variables in the model. Models for post-transport TUS and CRS used a multinomial distribution. We analyzed blood variables using a normal distribution, except for IG, which failed normality tests on the model residuals, and was subsequently analyzed using log-transformed variables. We used least square means with Tukey adjusted *p*-values to assess pairwise comparisons for TUSpre and CRSpre.

We used the margins function within SAS PROC GLIMMIX to calculate estimated marginal means and confidence limits for all fixed effects. Due to model constraints, we obtained estimated marginal means for TUSmax and CRSmax using Gaussian distributions. We used model predicted values to graph plots with trendlines for continuous variables of age and body weight.

### 2.5 Ethical approval

The experiment was approved by the Teagasc Animal Ethics Committee (Fermoy, Ireland, Approval number: TAEC2021-326) and the Health Products Regulatory Authority (Dublin, Ireland, Approval number: AE19132/P154).

## 3 Results

**Figure 2:**
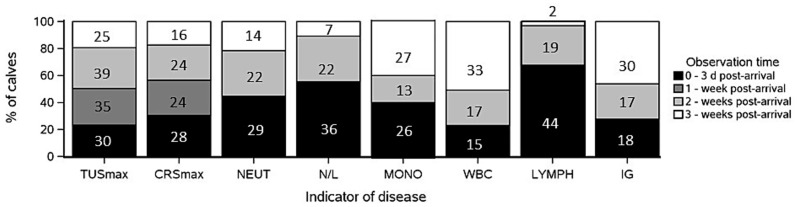
Stacked bar chart showing the observation time (0–3 days post-arrival, approximately 1-week post arrival, 2-weeks post arrival, and 3-weeks post arrival) at which calves reached the highest or lowest value for each health indicator. Each bar represents one indicator [TUSmax, CRSmax, highest recorded neutrophil (NEUT), N/L ratio (N/L), monocyte (MONO), and white blood cell counts (WBC), or lowest recorded lymphocyte count (LYMPH) or total immunoglobulins (IG)]. The stacked segments reflect the percentage of calves affected at each observation time. Numbers within bars represent number of calves. For TUSmax and CRSmax, the total number of observations exceeds 65 calves, as some calves reached equally high values at multiple observation times.

For individual calves, the highest or lowest value for indicators of respiratory disease between arrival and 3-weeks post-transport occurred at different observation times (Figure 2). The highest neutrophil count, N/L ratio, and lowest lymphocyte count were most frequently observed between 0- and 3-days post arrival. In contrast, the highest recorded monocyte count and white blood cell count were most often observed at 3-weeks post arrival, similar to the lowest recorded IG concentration. For categorical variables of TUSmax and CRSmax, it was common for calves to have equally high values across more than one observation time. On average, the highest observed TUSmax and CRSmax occurred at the earlier stages of the study. The highest observed TUS was a score of 4, which occurred once in three individual calves, one on arrival, one approximately 2-weeks post arrival, and one approximately 3-weeks post arrival. On arrival and two-weeks post arrival, 9 calves presented with a TUS of 3 or greater. Two calves were observed with a CRS of 7, at 1- and 2-weeks post arrival, respectively. A CRS of 6 was observed in four calves, one at 1-week, one at 2-weeks post arrival, and two calves at 3-weeks post arrival.

### 3.1 Precipitating factors

#### 3.1.1 Descriptive statistics

**Figure 3:**
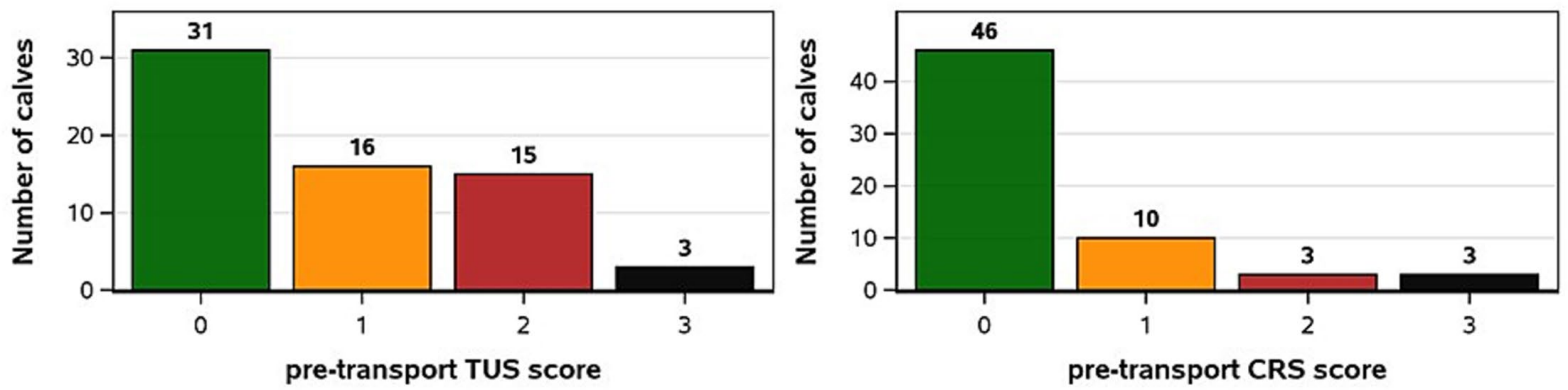
Frequencies of thoracic ultrasound score (TUS) and clinical respiratory score (CRS) severities observed pre-transport.

The frequencies of pre-transport TUS and CRS scores are shown in Figure 3. For pre-transport TUS, 31 calves had a score of 0. Sixteen calves had a score of 1: 10 of which showed comet-tail artefacts only in the cranial aspect of the right lung, one had artefacts solely in the caudal aspect of the right lung, one had artefacts solely in the caudal aspect of the left lung, and three had artefacts in both the cranial and caudal aspects of the right lung. One additional calf showed minor comet-tail artefacts in both cranial and caudal aspects of both lungs. Fifteen calves had a pre-transport TUS of 2, of these, nine had abnormalities in the cranial aspect of the right lung, one in the cranial aspect of the left lung, one in the caudal aspect of the right lung, three in the cranial aspects of both lungs, and one had abnormalities in both the cranial and caudal aspects of the right lung as well as the cranial aspect of the left lung. For all calves with a pre-transport TUS of 2 and multiple affected locations, the total consolidation equaled less than one lobe of total functional lung loss. A severe pre-transport TUS (score of 3) was observed in three calves, all of which were the effect of consolidation in the cranial aspect of the right lung.

Signs of precipitating disease based on pre-transport CRS were less common. Forty-six calves had a total pre-transport CRS of 0, showing no eye or nose discharge, coughing, or abnormal ear position. Ten calves had a pre-transport CRS of 1: seven of which had a small amount of eye discharge, one had a small amount of nose discharge, one had abnormal ear position, and one had an occasional cough. Three calves had a pre-transport CRS of 2, all were caused by abnormal eye discharge. Lastly, three calves had a pre-transport CRS of 3, two showed heavy ocular discharge, and the last had a slight unilateral ear droop as well as an occasional cough.

#### 3.1.2 Pre-transport TUS and CRS

**Figure 4:**
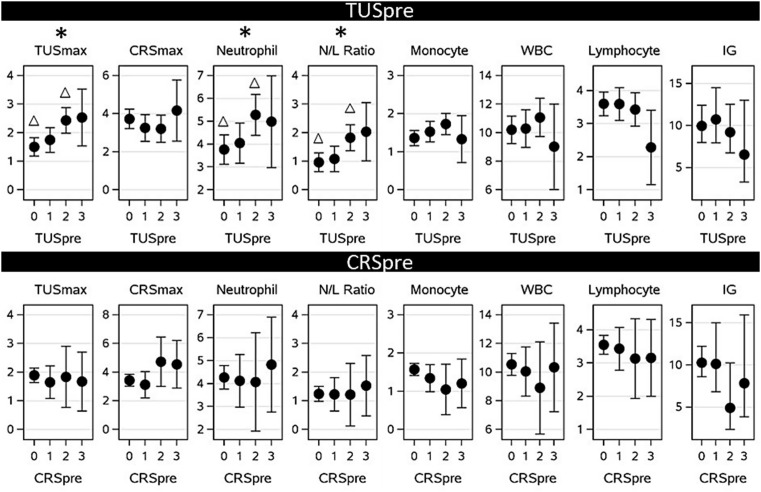
Associations between precipitating factors (pre-transport thoracic ultrasound score: TUS_pre_; 0, 1, 2, or 3, and pre-transport clinical respiratory score: CRS_pre_; 0, 1, 2, or 3) on marginal estimates (± confidence limits) of post-transport health measured as the highest thoracic ultrasound score (TUS_max_), clinical respiratory score (CRS_max_), neutrophil count, monocyte count, white blood cell count (WBC) (all in 10^9^/L), neutrophil:lymphocyte ratio (N/L Ratio), or the lowest lymphocyte count (in 10^9^/L) or total immunoglobulins (IG in g/L) observed between arrival and 3 weeks post arrival after calves were transported from Ireland to The Netherlands. * Significant difference (*p* = 0.05). Within graphs, columns marked with a ∆ differ significantly based on Tukey-adjusted post-hoc analysis (*p* = 0.05).

The effects of pre-transport TUS and pre-transport CRS on post-transport likelihood of developing signs of respiratory disease as determined by TUSmax, CRSmax and immunity-based blood variables are presented in Figure 4. In the case of TUSmax, the results indicate that a calf with a pre-transport TUS of 0 is likely to reach a lower post-transport TUSmax than a calf with a pre-transport TUS of 2 (*p* < 0.01). Calves with a pre-transport TUS of 0 and 1 were estimated to experience a TUSmax of 1.50 (SE: 0.159; 95% CI: 1.182–1.821) and 1.74 (SE: 0.217; 95% CI: 1.308–2.180) respectively, indicating mild to moderate lesions, while calves with a pre-transport TUS of 2 showed a TUSmax of 2.43 (SE: 0.222; 95% CI: 1.985–2.878), indicating moderate to severe thoracic lesions.

Pre-transport TUS had no association with CRSmax at any level (*p* = 0.88). In the case of blood immune variables, the highest observed neutrophil count and N/L ratio between arrival and 3-weeks post arrival were higher for a calf with a pre-transport TUS of 2 than for a calf with a pre-transport TUS of 0 (*p* = 0.04 and *p* = 0.02 respectively). Calves with a pre-transport TUS of 0 were expected to reach peak neutrophil counts and N/L ratios of 3.8 10^9^/L (SE: 0.321; 95% CI: 3.123–4.411) and 0.96 (SE: 0.163; 95% CI: 0.635–1.290) respectively, while calves with a pre-transport TUS of 2 were expected to reach peak values of 5.3 10^9^/L (SE: 0.448; 95% CI: 4.381–6.181) and 1.82 (SE: 0.228; 95% CI: 1.356–2.281) respectively. The highest observed monocyte count and WBC and the lowest observed lymphocyte count and IG between arrival and 3-weeks post arrival were not affected by pre-transport TUS (*p* = 0.18, *p* = 0.58, *p* = 0.17 and *p* = 0.58 respectively). No variables were affected by pre-transport CRS (CRSpre) (all *p* > 0.2).

### 3.2 Predisposing factors

#### 3.2.1 Breed and sex

**Figure 5:**
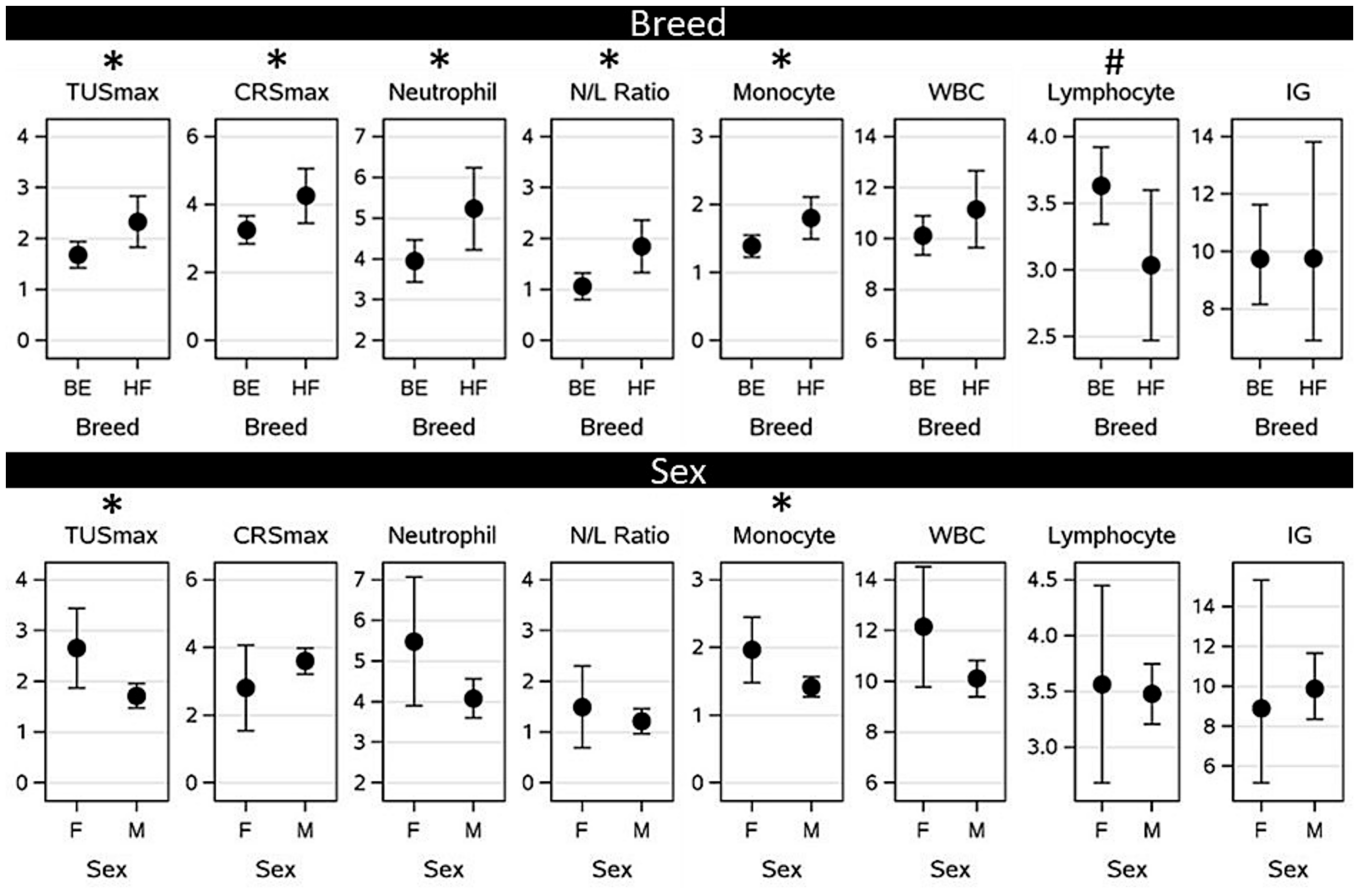
Associations between predisposing factors [breed: Holstein-Friesian (HF) or HF*Beef (BE), and sex: male (M) or female (F)] on post-transport health measured as the highest thoracic ultrasound score (TUS), clinical respiratory score (CRS), neutrophil count, monocyte count, white blood cell count (WBC) (all 10^9^/L), Neutrophil: lymphocyte ratio (N/L ratio), or the lowest lymphocyte count (in 10^9^/L) or total immunoglobulins (IG in g/L) observed between arrival and 3 weeks post-arrival after calves were transported from Ireland to the Netherlands. * Significant difference (*p* = 0.05), # tendency to differ (*p* = 0.1).

The effects of breed and sex on post-transport health as determined by TUSmax, CRSmax and immunity-based blood variables are presented in Figure 5. The study included Holstein-Friesian (*n* = 15) and Holstein-Friesian × Beef (beef-cross; *n* = 50) calves, and, on average, TUSmax, CRSmax, and the highest observed neutrophil count, N/L ratio and monocyte count between arrival and 3-weeks post arrival were higher for Holstein-Friesian than for beef-cross calves (*p* = 0.02, *p* = 0.01, *p* = 0.04, *p* = 0.01, and *p* = 0.03 respectively). Beef-cross calves were estimated to have a TUSmax and CRSmax of 1.68 (SE: 0.128; 95% CI: 1.425–1.938) and 3.26 (SE: 0.206; 95% CI: 2.844–3.671) respectively, versus 2.33 (SE: 0.249; 95% CI: 1.831–2.832) and 4.26 (SE: 0.402; 95% CI: 3.452–5.068) in Holstein Friesian calves. Neutrophil count, N/L ratio, and monocyte count were estimated to be 3.95 10^9^/L (SE: 0.257; 95% CI: 3.438–4.471), 1.06 (SE: 0.131; 95% CI: 0.799–1.325), and 1.39 10^9^/L (SE: 0.079; 95% CI: 1.231–1.549) for beef-cross calves and 5.23 (SE: 0.503; 95% CI: 4.22–6.24), 1.85 (SE: 0.256; 95% CI: 1.340–2.365), and 1.81 (0.154; 95% CI: 1.495–2.116) for Holstein-Friesian calves, respectively. Breed had no effect on the highest observed WBC or lowest observed lymphocyte count or IG between arrival and 3-weeks post arrival (*p* = 0.19 and *p* = 0.40 and *p* = 0.89 respectively).

The study included 56 male and 9 female calves, on average, TUSmax and the highest recorded monocyte count between arrival and 3-weeks post arrival were, on average, higher for female than for male calves (*p* = 0.02 and *p* = 0.05 respectively). TUSmax was estimated to be 2.66 (SE: 0.390; 95% CI: 1.874–3.443) for female calves and 1.72 (SE: 0.119; 95% CI: 1.478–1.956) for male calves. Monocyte count was estimated to be 1.97 10^9^/L (SE: 0.242; 95% CI: 1.479–2.451) for female calves and 1.42 10^9^/L (SE: 0.074; 95% CI: 1.272–1.568) for male calves. Sex did not affect CRSmax (*p* = 0.51), the highest recorded neutrophil count (*p* = 0.11), N/L ratio (*p* = 0.53), WBC (*p* = 0.12), or the lowest recorded lymphocyte count (*p* = 0.86) or IG (*p* = 0.73).

#### 3.2.2 Transport cohort and source

**Figure 6:**
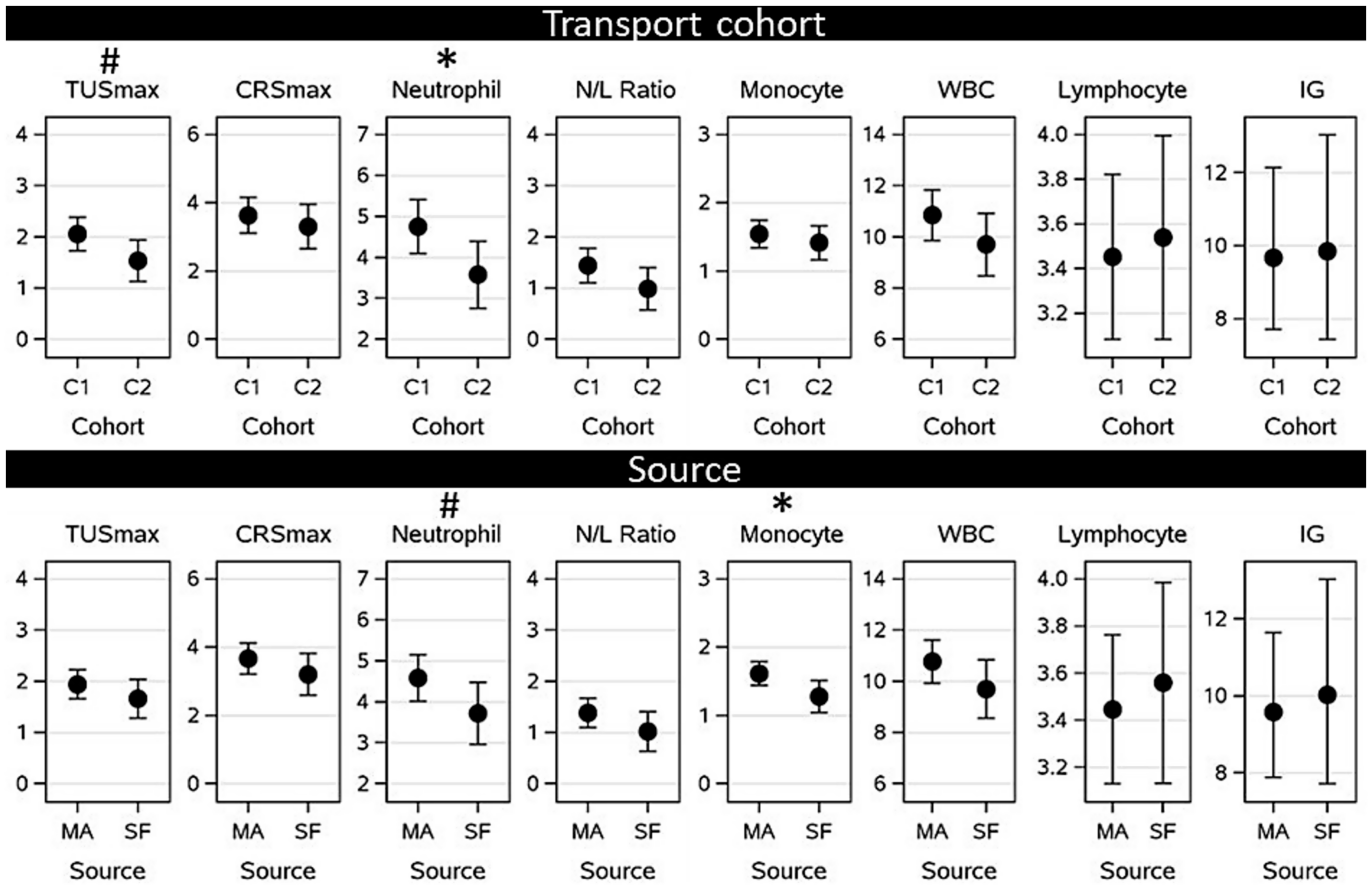
Associations between predisposing factors (transport cohort 1 [C1] and 2 [C2], and source: dairy source farm [SF] or livestock mart [MA]) on post-transport health measured as the highest thoracic ultrasound score (TUS), clinical respiratory score (CRS), neutrophil count, monocyte count, white blood cell count (WBC) (all 10^9^/L), neutrophil: lymphocyte ratio (N/L ratio), or the lowest lymphocyte count (in 10^9^/L) or total immunoglobulins (IG in g/L) observed between arrival and 3 weeks post-arrival after calves were transported from Ireland to The Netherlands. * Significant difference (*p* = 0.05), # tendency to differ (*p* = 0.1).

The effects of transport cohort and source on post-transport health as determined by TUSmax, CRSmax and immunity-based blood variables are presented in Figure 6. In terms of transport cohort, the highest observed neutrophil count between arrival and 3-weeks post arrival was higher for calves transported in cohort 1 than for calves transported in cohort 2 (*p* = 0.05). Calves in transport cohort 1 were estimated to reach a highest recorded neutrophil count of 4.76 10^9^/L (SE: 0.328; 95% CI: 4.099–5.418) versus 3.58 (SE: 0.407; 95% CI: 2.762–4.397) for calves in transport cohort 2. Transport cohort had no effect on the highest observed TUSmax (*p* = 0.10), CRSmax (*p* = 0.30), N/L ratio (*p* = 0.14), monocyte count (*p* = 0.49), and white blood cell count (*p* = 0.21), or the lowest recorded lymphocyte count (*p* = 0.80) and IG (*p* = 0.93) between arrival and 3-weeks post arrival.

For source, the highest recorded monocyte count between arrival and 3-weeks post arrival was higher for mart calves than for source farm calves (*p* = 0.03). Mart calves were estimated to reach monocyte counts of 1.62 10^9^/L (SE: 0.087; 95% CI: 1.442–1.790) versus 1.28 10^9^/L (SE: 0.117; 95% CI: 1.043–1.512) in source farm calves. The source of the calf had no effect on the highest recorded TUSmax (*p* = 0.14), CRSmax (*p* = 0.12), neutrophil count (*p* = 0.09), N/L ratio (*p* = 0.16), and WBC (*p* = 0.16) and the lowest recorded lymphocyte count (*p* = 0.69) and IG (*p* = 0.79).

#### 3.2.3 Age and body weight at departure

**Figure 7:**
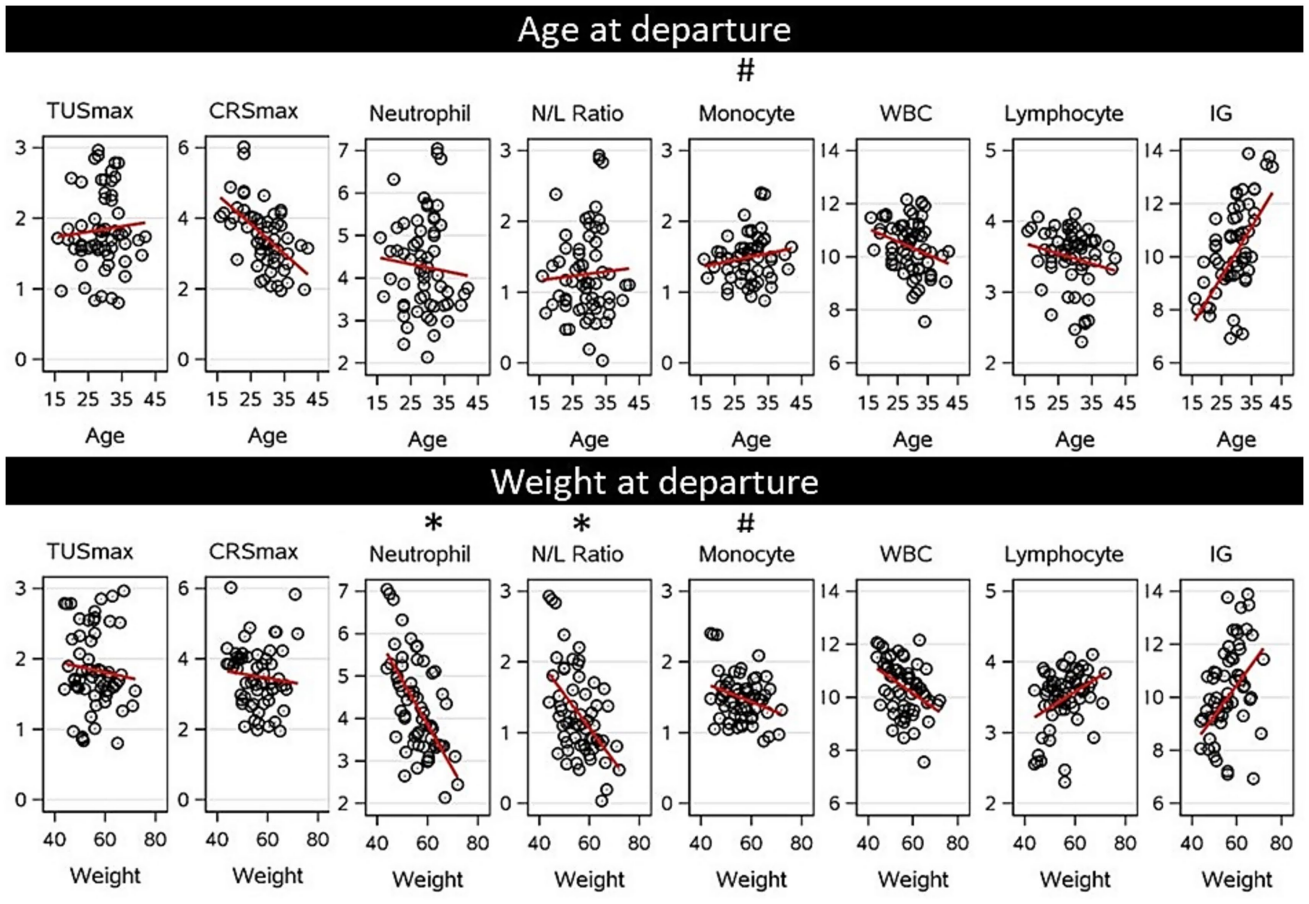
Effect of the age at departure (16–42 d) on post-transport health measured as the highest thoracic ultrasound score (TUS), clinical respiratory score (CRS), neutrophil count, monocyte count, white blood cell count (WBC) (all 10^9^/L), neutrophil:lymphocyte ratio (N/L ratio), or the lowest lymphocyte count (in 10^9^/L) or total immunoglobulins (IG in g/L) observed between arrival and 3 weeks post arrival after calves were transported from Ireland to the Netherlands. A linear trendline indicates the predicted relationship between age or body weight at departure and each health outcome. * Significant relationship (*p* = 0.05), # tendency of relationship (*p* = 0.1).

The relationship between age and body weight at departure and post-transport health measured by TUS, CRS and blood variables indicative of the immune status are presented in Figure 7. On average, calves were 29.1 d old at departure, ranging from 16 to 42 d, they weighed 56.1 kg on average, ranging from 43 to 73 kg at departure. There was no association between maximum TUS (*p* = 0.48), CRS (*p* = 0.86) or the remaining blood-based immune cell counts with age, including the highest recorded WBC (*p* = 0.79), neutrophil count (*p* = 0.63), N/L ratio (*p* = 0.24), monocyte count (*p* = 0.09), or the lowest recorded lymphocyte count (*p* = 0.20) or IG (*p* = 0.79).

However, significant associations were observed between body weight and neutrophil associated variables. The highest observed neutrophil count and N/L ratio between arrival and 3-weeks post arrival were higher for calves that weighed less at departure (both *p* < 0.01). The highest observed neutrophil count decreased by 0.103 10^9^/L (± 0.0336 10^9^/L) for every 1 kg increase in body weight at the point of departure. The highest observed N/L ratio decreased by 0.048 (± 0.0171) for every 1 kg of additional body weight at departure. The calf’s body weight at departure did not affect the highest observed post-transport TUSmax (*p* = 0.48), CRSmax (*p* = 0.86), WBC (*p* = 0.30), and monocyte count (*p* = 0.10) or the lowest observed lymphocyte count (*p* = 0.13) and IG (*p* = 0.36) between arrival and 3-weeks post arrival.

### 3.3 Summary

A summary of risk factor associations with the highest and lowest values of variables indicative of respiratory disease is presented in Table 2. In general, breed significantly affected five out of eight post-arrival variables, while pre-transport TUS affected three variables, sex and weight affected two variables each, and cohort and source affected one variable each. Pre-transport CRS and age did not significantly affect any variables indicative of the development of respiratory disease post-transport.

**Table 2:** Associations [significances (*p = 0.05; shaded green) and tendencies (#*p* = 0.10; shaded orange)] between pre-transport precipitating and predisposing factors and post-transport respiratory disease signs.

Risk factors	Post-transport variables indicative of respiratory disease							
	TUSmax	CRSmax	Neutrophil	N/L ratio	Monocyte	WBC	Lymphocyte	IG
TUSpre	*		*	*				
CRSpre								
Breed	*	*	*	*	*		#	
Sex	*				*			
Cohort	#		*					
Source			#		*			
Age					#			
Weight			*	*	#			

Post-transport variables indicative of respiratory disease represent either the highest (TUSmax, CRSmax, neutrophil, N/L ratio, Monocyte and WBC count) or lowest (Lymphocyte count and IG) recorded value between arrival and 3-weeks post arrival.

## 4 Discussion

We aimed to examine the association between pre-transport risk factors and the post-transport likelihood of signs of respiratory disease based on thoracic ultrasound, clinical assessment and blood variables, in calves deemed legally fit for transport. In interpreting our findings, it is important to note that thoracic ultrasound scoring (TUS) and clinical respiratory scoring (CRS) are not specifically diagnostic in nature, but farmers and veterinarians use them routinely as indicators of respiratory pathology or clinical signs thereof. We therefore used them as indicators of respiratory infection, regardless of the cause, with higher scores reflecting more severe lesions or more clinical signs of disease. Blood cell analysis is more complex to interpret, as values represent a balance between production and consumption. Respiratory disease typically leads to lymphopenia, monocytosis, neutrophilia, a higher neutrophil-to-lymphocyte (N/L) ratio, reduced immunoglobulins, and/or an increased WBC count (7, 13). However, stress, such as experienced during long-distance transport, as well as other infections, may impact blood indicators of disease, confounding the direction of change and potentially leading to no change at all (2, 26). This complexity means that changes in immune cells cannot be interpreted unambiguously, but as they are not subject to observer bias and can reveal subtle changes in immune function, their inclusion still adds value to respiratory disease detection.

Because we used the most extreme (highest or lowest) value for each calf within the first 3 weeks post-arrival rather than analyzing specific intermediate time points, patterns of disease progression over time were not evaluated. Additionally, we performed post-transport health assessments at regular intervals, but not daily, therefore the most severe respiratory health events may have gone undiagnosed in some cases, affecting maximum recorded scores used in the analysis. We also collected thoracic ultrasound scoring and clinical respiratory scoring data under suboptimal conditions. Calves were housed individually on-farm, which made thoracic ultrasound observations difficult due to limited space for the observer in the same pen as the calf, while pre-transport CRS did not include rectal temperatures due to practical constraints, which may have reduced the detection of early signs of viral infections. Lastly, no pre-transport management data was available for this trial, and therefore information on colostrum feeding, feeding management, prior disease, or additional prior transport was unknown and unfortunately these factors could not be accounted for in the results.

### 4.1 Precipitating factors

In this study, we regarded prior or ongoing subclinical respiratory infection as precipitating risk factor, directly triggering respiratory disease leading to higher pre-transport TUS or CRS values. As a predictor of signs of post-transport respiratory disease development, pre-transport TUS demonstrated a more consistent relationship, whereby calves that were transported with a TUS of 2 had a significantly greater maximum TUS score after arrival relative to calves that commenced their journey with a TUS of 0. It was also evident, and noteworthy, that of those 31 calves with a pre-transport TUS of 0, only 13% maintained this score, despite all calves having repeated antibiotic treatment. This suggests that transport and associated stressors were associated with the development of lung lesions in calves that were initially free of detectable respiratory abnormalities and that antibiotic treatments alone were insufficient to mitigate the impact of transport-related respiratory disease development. Pre-transport TUS was also significantly associated with neutrophil count and N/L ratio, with higher maximum values for calves with high pre-transport TUS values. Widely regarded as a primary mediator of innate immunity, and the most abundant leukocytes in the circulation, the neutrophil correlation with increasing lung pathology is understandable. The lack of association with other immune blood cells may be more reflective of their location, and function in areas beyond the circulatory blood pool.

The lack of association between pre-transport TUS and post-transport maximum CRS values might be due to these methods screening for different pathology. While TUS allows detection of lesions in deeper lung tissue, CRS often reflects upper respiratory infections (9) but may also identify early viral infections before any lung pathology is evident via TUS. Thoracic ultrasound scoring may also show consolidations that are not actively infected but rather have been unable to fully heal.

In contrast to pre-transport TUS, pre-transport CRS was not a reliable predictor of post-transport respiratory disease. However, limitations such as the lack of rectal temperature collection pre-transport and small sample size must be considered when interpreting the results. Results in this study demonstrated no significant relationship between pre-transport CRS and any of the post-transport variables investigated. This tentatively confirms the limited sensitivity of CRS (9). Clinical respiratory scoring can be considered subjective, relying heavily on assessor experience, and observer reliability is often poor (27), which may further reduce its predictive accuracy. Clinical respiratory scoring may not always detect early stages of respiratory disease, meaning calves with internal lung inflammation or damage, but without clinical symptoms, can still be deemed fit for transport (28). These factors suggest that while CRS remains practical and widely used on farms, its ability to identify calves with subclinical respiratory disease may be limited compared to TUS.

The conclusion in this instance is that prior or ongoing respiratory infection can be reliably assessed by TUS but not by CRS. As a screening tool, CRS is extensively used to assess fitness for transport such as by veterinarians to meet legal requirements, including in this study. Further tightening of CRS cut-offs, even confining fitness for transport to those who score a 0 would not seem to benefit calf selection. However, our results suggest the potential benefit of expanding calf health screening to include TUS, perhaps in addition to CRS, prior to transport. Our findings suggest that calves with a pre-transport TUS of 2 or higher may be more prone to negative health outcomes post transport, although additional research is needed to confirm this cut-off value. Tightening fitness for transport legislations may deem more calves unfit for transport. Calves deemed unfit for transport will have to remain at the assembly center until they recover, or be sold locally, avoiding negative welfare consequences of transporting already diseased calves. TUS assessments require additional time, but quick sampling techniques, such as used by Jourquin et al. (29), might improve the efficiency of using TUS in pre-transport and post-transport respiratory health assessments.

### 4.2 Predisposing factors

In this study, we regarded environmental or genetic factors as predisposing risk factors, including breed, sex, transport cohort, source, and age and body weight at departure. Among these factors, breed showed the clearest association with signs of respiratory disease development. Holstein-Friesian calves demonstrated increased susceptibility, with more pronounced clinical and physiological signs relative to beef-cross calves. Holstein-Friesian calves are known to develop more severe signs of respiratory disease than other dairy breeds (30), while calves crossed between two beef breeds may have a lower risk of developing respiratory disease than purebred beef calves (31). However, little is known about the effects of crossbreeding dairy cows with beef sires on respiratory outcomes. Our findings suggest that using beef sires in dairy systems may slightly reduce respiratory disease in calves exposed to commingling, transport, and rearing in a white veal system.

Regarding pre-transport body weight, lighter calves showed more signs of respiratory disease between arrival and 3-weeks post arrival. Calf body weight is widely recognized as an indicator of both fitness for transport and post-transport disease risk, with lighter calves consistently more vulnerable (32–34). In Ireland, calves must weigh at least 40 kg to be deemed suitable for transport, but research suggests a minimum of 50 kg is more appropriate (35). Among our study calves, 13 out of 65 weighed less than 50 kg. Tightening body weight thresholds could prevent these lighter calves from leaving the dairy farm, preventing unnecessary transport of unfit calves and reducing post-transport respiratory disease prevalence. In our study, beef-cross calves were generally heavier, but 8 out of 13 calves under 50 kg at departure were of beef cross composition. Body weight is preferred as a proxy for transport fitness compared to other factors such as age, as body weight is more reliably measured and enforceable (35). In line with this, the clearer association between body weight and post-transport disease in our study supports prioritizing body weight over age as a predictive risk factor.

Cohort, sex, and source showed weaker or inconsistent associations with respiratory disease development. While calves transported in transport cohort 1, female calves, and those originating from a livestock mart exhibited more signs of respiratory disease post-transport, these trends were minor and lacked consistency. Sex-based effects in disease outcomes are not necessarily expected (36, 37), especially in our study in which the group of female calves consisted of just nine animals, and all were beef-cross calves. Transport cohorts face varying transport conditions, including transport duration, feed withdrawal durations, or extreme weather events, which may account for some variation (22, 38, 39). While transport cohort 2 calves experienced a longer total journey duration and more severe weather during sea transport than their counterparts in transport cohort 1, surprisingly, respiratory disease outcomes were worse (though minimally) in cohort 1 calves. These cohort-specific effects suggest that transport variability can influence post-transport physiology, although its specific impact on respiratory disease was less pronounced, and the direct cause of variation is unknown. The lack of clear differences related to these factors was partially due to a lack of statistical power, but may also reflect subtle effects or the influence of multiple interacting factors, rather than an absence of biological relevance.

Calves sourced from livestock marts have previously been observed to differ physiologically from those sourced directly from dairy farms, although differences were limited to the point of origin (22). Purchasing calves through livestock marts and the associated commingling is generally viewed as a risk factor for respiratory diseases (34). However, in the present study, dairy farm-sourced calves were also commingled at several points along their journey, which may have offset the advantages of direct sourcing. Although mart calves exhibited a monocytosis during the recovery period, this difference was minimal. Patterns linked to source were only evident in rapidly changing indicators (blood variables), and even these were minor. Overall, there was no clear evidence that source influenced disease development in this system.

Calf age is often debated in discussions of potential transport legislation. Older calves may show fewer post-transport respiratory disease signs (20, 36), but the effect is often so small it is not worth reporting (40). The recorded age may also be inaccurate, particularly in spring-calving systems like Ireland, where farmers often register a cohort of calves but must wait for tagging until after birth, or occasionally waiting several days so multiple calves can be registered and tagged at once. While age offers some indication of development, such as immune system maturity (41), it is a poor proxy for health status, particularly for calves that receive insufficient colostrum or that fail to sufficiently grow in the first weeks after birth and therefore may have low energy reserves to cope with transport stressors. Our study found no clear association between age at transport and post-transport disease signs, while the sample size was small, this may give some further confirmation that age is a poor proxy for post-transport success, especially when compared to using weight at departure as a risk factor.

### 4.3 Indicator relevance for detecting post-transport respiratory disease

Out of eight variables included to determine post-transport health, five demonstrated associations with risk factors. We considered variables relevant if they associated with precipitating or predisposing risk factors. In particular, the highest recorded neutrophil count, which was associated with four predisposing factors. Other relevant post-transport health variables included thoracic ultrasonography, monocyte count, and N/L ratio (each affected by three factors). While a greater number of associations may suggest that a variable is more reflective of respiratory disease outcomes, this does not necessarily indicate diagnostic value or specificity. Some variables may be more responsive to infection but less specific, particularly blood-related variables which may change rapidly. Other variables, such as thoracic ultrasound scores, may show fewer associations, but offer more targeted insights into calf respiratory health.

Interestingly, post-transport thoracic ultrasound and N/L ratio displayed a near identical profile in relation to most risk factors in our study, particularly for pre-transport TUS. Post-transport clinical respiratory scores were only affected by breed while white blood cell count, lymphocyte count, and IG were never significantly affected. Taken together, these findings suggest that further research focusing on thoracic ultrasound and N/L ratio as risk factors for future respiratory disease would be of greater value.

Post transport disease detection in a veal system is complicated. Clinical signs of respiratory disease are most commonly used, but in this study, the farmers and their veterinarians treated all calves with antibiotics, which may have compromised the value of post-transport clinical assessments for respiratory disease detection (42). Since blood samples are routinely collected on veal farms to determine hemoglobin concentrations, they may also serve as a tool for respiratory screening. Although we must assess the cost-to-benefit ratio on a case-by-case basis, routine screening may identify calves which require closer monitoring. In this study, blood samples were valuable for detecting differences related to risk factors. In all cases where the N/L ratio was impacted by a risk factor, neutrophil count was also affected. Monocyte count additionally revealed differences associated with sex and source. Using neutrophil and monocyte counts may therefore be useful for screening respiratory disease in the future.

Post-transport thoracic ultrasound scoring was also valuable; producing consistent results with neutrophil count for the risk factors of pre-transport TUS, breed, and cohort, while pre-transport TUS aligned with monocyte count for breed and sex. Depending on the number of animals screened and the practical limitations, blood samples or TUS could be selected as tools for post-transport respiratory disease detection.

### 4.4 Legislative enhancements

Continual improvement in legislation controlling the welfare of animals during transport is necessary. Within the European Union, for example, current legislation mandates fitness-for-transport assessments prior to long-distance transport [EC 1/2005; (4)], however, this approach could be improved. Current requirements ensure that animals are at least 14 days old and have received colostrum, but these factors are often difficult to verify, and no specific (clinical) assessment methods are mandated. Our study suggests that CRS underperforms compared to TUS, indicating that EU legislation could be strengthened by specifying which assessment tools are sufficient to identify at-risk calves prior to transport, however, further research is required to fully evaluate the efficacy of TUS, CRS, and other potential assessment methods. Anticipated legislation in the European Union is expected to include minimum weight requirements (likely 50 kg) and to raise the minimum transport age to 28 days. The results of our study suggest that placing a greater emphasis on weight rather than age is likely to more effectively reduce respiratory disease in calves after long-distance transport. As weight is also easier to verify than age, this approach would enable a more reliable selection of calves fit for long-distance transport.

## 5 Conclusion

This study found that on average, the likelihood of a calf not succumbing to respiratory disease following transport is significantly augmented for a heavy male beef-cross calf with a pre-transport thoracic ultrasound score of 0, mindful of transport weather and source, and irrespective of age and clinical respiratory score. Clinical respiratory scoring at the point of departure underperformed as a predictor of post-transport health, especially when compared to pre-transport thoracic ultrasound scoring, though further research is required to assess the full efficacy of these and other factors on long-term health at the destination farm. On the other hand, pre-transport TUS partially predicted post-transport respiratory health and should be considered as a practical pre-transport screening tool to enhance the health of calves in transport. We suggest veterinarians and other personnel performing fitness-for-transport assessments should implement pre-transport TUS assessments to identify at-risk animals. Furthermore, policymakers should consider updating guidelines on fitness-for-transport assessments, either by incorporating TUS requirements or by supporting the development of other novel screening systems.

## Data Availability

The original contributions presented in the study are included in the article/Supplementary material, further inquiries can be directed to the corresponding author.

## References

[1] Pardon B De Bleecker K Hostens M Callens J Dewulf J Deprez P. Longitudinal study on morbidity and mortality in white veal calves in Belgium. BMC Vet Res. (2012) 8:1–15. doi: 10.1186/1746-6148-8-26 22414223 PMC3366893

[2] Earley B Buckham Sporer K Gupta S. Invited review: relationship between cattle transport, immunity and respiratory disease. Animal. (2017) 11:486–92. doi: 10.1017/S1751731116001622, 28209213

[3] Wilcox C Schutz M Rostagno M Lay D Jr Eicher S. Repeated mixing and isolation: measuring chronic, intermittent stress in Holstein calves. J Dairy Sci. (2013) 96:7223–33. doi: 10.3168/jds.2013-6944, 24054297

[4] European Parliament. Council regulation (EC) no 1/2005: on the protection of animals during transport and related operations. Council of the European Union. (2005).

[5] Buczinski S Pardon B. Bovine respiratory disease diagnosis: what progress has been made in clinical diagnosis? Vet Clin. (2020) 36:399–423. doi: 10.1016/j.cvfa.2020.03.004, 32451033

[6] Ollivett TL Buczinski S. On-farm use of ultrasonography for bovine respiratory disease. Vet Clin N Am Food Anim Pract. (2016) 32:19–35. doi: 10.1016/j.cvfa.2015.09.001, 26922110

[7] Pardon B Alliët J Boone R Roelandt S Valgaeren B Deprez P. Prediction of respiratory disease and diarrhea in veal calves based on immunoglobulin levels and the serostatus for respiratory pathogens measured at arrival. Prev Vet Med. (2015) 120:169–76. doi: 10.1016/j.prevetmed.2015.04.009, 25937168 PMC7114331

[8] Šoltésová H Nagyová V Tóthová C Nagy O. Haematological and blood biochemical alterations associated with respiratory disease in calves. Acta Vet Brno. (2015) 84:249–56. doi: 10.2754/avb201584030249

[9] Berman J. Literature review of the principal diagnostic tests to detect bovine respiratory disease in pre-weaned dairy and veal calves. Animals. (2024) 14:329. doi: 10.3390/ani14020329, 38275791 PMC10812408

[10] Anwar MR Abd El-Raof YM El-Attar HM Hefnawy AE Ghanem MM. Evaluation of clinical and hematobiochemical alterations in naturally occurring bovine respiratory disease in feedlot cattle calves in Egypt. Benha Vet Med J. (2019) 36:305–13. doi: 10.21608/bvmj.2019.16753.1088

[11] Fraser BC Anderson DE White BJ Miesner MD Lakritz J Amrine D . Associations of various physical and blood analysis variables with experimentally induced *Mycoplasma bovis* pneumonia in calves. Am J Vet Res. (2014) 75:200–7. doi: 10.2460/ajvr.75.2.200, 24471757

[12] Coskun A Guzelbektes H Simsek A Aydogdu U Sayin Z Sen I. Rev Med Vet. (2012) 163:615–20.

[13] Moisá SJ Aly SS Lehenbauer TW Love WJ Rossitto PV Van Eenennaam AL . Association of plasma haptoglobin concentration and other biomarkers with bovine respiratory disease status in pre-weaned dairy calves. J Vet Diagn Invest. (2019) 31:40–6. doi: 10.1177/1040638718807242, 30328386 PMC6505765

[14] Berman J Francoz D Dufour S Buczinski S. Bayesian estimation of sensitivity and specificity of systematic thoracic ultrasound exam for diagnosis of bovine respiratory disease in pre-weaned calves. Prev Vet Med. (2019) 162:38–45. doi: 10.1016/j.prevetmed.2018.10.025, 30621897

[15] Loneragan GH Dargatz DA Morley PS Smith MA. Trends in mortality ratios among cattle in US feedlots. J Am Vet Med Assoc. (2001) 219:1122–7. doi: 10.2460/javma.2001.219.1122, 11700712

[16] Renaud D Waalderbos K Beavers L Duffield T Leslie K Windeyer M. Risk factors associated with failed transfer of passive immunity in male and female dairy calves: a 2008 retrospective cross-sectional study. J Dairy Sci. (2020) 103:3521–8. doi: 10.3168/jds.2019-17397, 32037177

[17] Barry J Bokkers EA Berry D de Boer IJ McClure J Kennedy E. Associations between colostrum management, passive immunity, calf-related hygiene practices, and rates of mortality in preweaning dairy calves. J Dairy Sci. (2019) 102:10266–76. doi: 10.3168/jds.2019-16815, 31521357

[18] Buckham-Sporer K Earley B Marti S. Current knowledge on the transportation by road of cattle, including unweaned calves. Animals. (2023) 13:3393. doi: 10.3390/ani13213393, 37958148 PMC10649969

[19] Renaud DL Duffield TF LeBlanc SJ Ferguson S Haley DB Kelton DF. Risk factors associated with mortality at a milk-fed veal calf facility: a prospective cohort study. J Dairy Sci. (2018) 101:2659–68. doi: 10.3168/jds.2017-13581, 29290439

[20] Goetz HM Creutzinger KC Kelton DF Costa JHC Winder CB Renaud DL. A randomized controlled trial investigating the effect of transport duration and age at transport on surplus dairy calves: part I. Impact on health and growth. J Dairy Sci. (2023) 106:2784–99. doi: 10.3168/jds.2022-22366, 36797186

[21] Marine Institute. Wave buoy observations (2022). Available online at: http://www.marine.ie/site-area/data-services/real-time-observations/wave-buoy-observations. (Accessed December 18, 2022).

[22] van Dijk LL Siegmann S Field NL Sugrue K van Reenen K Bokkers EAM . The effect of source and journey on physiological variables in calves transported by road and ferry between Ireland and the Netherlands. Front Vet Sci. (2023) 10:1238734. doi: 10.3389/fvets.2023.1238734 37829357 PMC10566621

[23] van Dijk LL Siegmann S Field NL Sugrue K van Reenen CG Bokkers EAM . Observational study: effect of varying transport durations and feed withdrawal on the physiological status and health of dairy calves. Ir Vet J. (2025) 78:1. doi: 10.1186/s13620-025-00287-2, 39806431 PMC11730127

[24] McGuirk SM Peek SF. Timely diagnosis of dairy calf respiratory disease using a standardized scoring system. Anim Health Res Rev. (2014) 15:145–7. doi: 10.1017/S1466252314000267, 25410122

[25] SAS Institute Incorporated. SAS/STAT® 15.2 User’s Guide. Cary, NC: SAS Institute Incorporated. (2024).

[26] Pardon B Callens J Maris J Allais L Van Praet W Deprez P . Pathogen-specific risk factors in acute outbreaks of respiratory disease in calves. J Dairy Sci. (2020) 103:2556–66. doi: 10.3168/jds.2019-17486, 31954585 PMC7094370

[27] Møller HH Krogh MA Petersen MB Nielsen LR Capion N. Comparison and interobserver reliability between a visual analog scale and the Wisconsin calf health scoring chart for detection of respiratory disease in dairy calves. J Dairy Sci. (2024) 107:1102–9. doi: 10.3168/jds.2023-23554, 37709013

[28] Hinnant HR Elder LA Claus-Walker R Mandella CM Slanzon GS Parrish LM . Comparative diagnoses of respiratory disease in preweaned dairy calves using sequential thoracic ultrasonography and clinical respiratory scoring. Aust Vet J. (2023) 102:187–99. doi: 10.1111/avj.13309, 38114290

[29] Jourquin S Lowie T Bokma J Pardon B. Accuracy and inter-rater agreement among practitioners using quick thoracic ultrasonography to diagnose calf pneumonia. Vet Rec. (2024) 194:no–o. doi: 10.1002/vetr.3896 38343074

[30] Johnson KF Chancellor N Wathes DC. A cohort study risk factor analysis for endemic disease in pre-weaned dairy heifer calves. Animals. (2021) 11:378. doi: 10.3390/ani11020378, 33540923 PMC7913234

[31] Snowder G Van Vleck LD Cundiff L Bennett G. Influence of breed, heterozygosity, and disease incidence on estimates of variance components of respiratory disease in preweaned beef calves. J Anim Sci. (2005) 83:1247–61. doi: 10.2527/2005.8361247x, 15890802

[32] Sanderson MW Dargatz DA Wagner BA. Risk factors for initial respiratory disease in United States’ feedlots based on producer-collected daily morbidity counts. Can Vet J. (2008) 49:373 18481546 PMC2275341

[33] Gummow B Mapham PH. A stochastic partial-budget analysis of an experimental *Pasteurella haemolytica* feedlot vaccine trial. Prev Vet Med. (2000) 43:29–42. doi: 10.1016/s0167-5877(99)00071-9, 10665949

[34] Taylor JD Fulton RW Lehenbauer TW Step DL Confer AW. The epidemiology of bovine respiratory disease: what is the evidence for predisposing factors? Can Vet J. (2010) 51:1095 21197200 PMC2942046

[35] Renaud D Pardon B. Preparing male dairy calves for the veal and dairy beef industry. Vet Clin. (2022) 38:77–92. doi: 10.1016/j.cvfa.2021.11.006, 35219487

[36] Marcato F van den Brand H Kemp B Engel B Schnabel SK Hoorweg FA . Effects of transport age and calf and maternal characteristics on health and performance of veal calves. J Dairy Sci. (2022) 105:1452–68. doi: 10.3168/jds.2021-20637, 34955258

[37] Hoffelner J Peinhopf-Petz W Wittek T. Associations between ultrasonographically diagnosed lung lesions, clinical parameters and treatment frequency in veal calves in an Austrian fattening farm. Animals. (2024) 14:2311. doi: 10.3390/ani14162311, 39199845 PMC11350914

[38] Bernardini D Gerardi G Peli A Nanni Costa L Amadori M Segato S. The effects of different environmental conditions on thermoregulation and clinical and hematological variables in long-distance road-transported calves. J Anim Sci. (2012) 90:1183–91. doi: 10.2527/jas.2011-4113, 22100587

[39] Phillips C. Transport of cattle, sheep and other livestock by sea and air In: Livestock handling and transport. GB: CABI (2024). 488–504.

[40] Santman-Berends I de Bont-Smolenaars AJG Roos L Velthuis AGJ van Schaik G. Using routinely collected data to evaluate risk factors for mortality of veal calves. Prev Vet Med. (2018) 157:86–93. doi: 10.1016/j.prevetmed.2018.05.013, 30086854 PMC7125930

[41] Hulbert LE Moisa SJ. Stress, immunity, and the management of calves. J Dairy Sci. (2016) 99:3199–216. doi: 10.3168/jds.2015-10198, 26805993

[42] Tomazi A Tomazi T Bringhenti L Vinhal A Rodrigues M Bilby T . Treatment with 2 commercial antibiotics reduced clinical and systemic signs of pneumonia and the abundance of pathogenic bacteria in the upper respiratory tract of preweaning dairy calves. J Dairy Sci. (2023) 106:2750–71. doi: 10.3168/jds.2022-22451, 36797182

